# Ultrasonographic assessment of bone erosions in the different subtypes of systemic lupus erythematosus arthritis: comparison with computed tomography

**DOI:** 10.1186/s13075-016-1125-8

**Published:** 2016-10-04

**Authors:** Matteo Piga, Luca Saba, Alessandra Gabba, Mattia Congia, Antonella Balestrieri, Alessandro Mathieu, Alberto Cauli

**Affiliations:** 1 Rheumatology Unit, University Clinic and AOU of Cagliari, Cagliari, Italy; 2 Department of Radiology, University of Cagliari, Cagliari, Italy; 3 Chair of Rheumatology and Rheumatology Unit, University Clinic AOU of Cagliari, Monserrato, CA SS 554-09042 Italy

**Keywords:** Systemic lupus erythematosus, Ultrasound, Computed tomography, Arthritis, Rhupus, Jaccoud’s arthropathy, Erosion

## Abstract

**Background:**

The aim was to determine the accuracy of high-resolution ultrasonography (US) for detecting erosion in the metacarpophalangeal (MCP) and wrist joints of patients with different subtypes of systemic lupus erythematosus (SLE) arthritis, using computed tomography (CT) as the gold-standard reference method.

**Method:**

The ulnar head, radiocarpal and second to fifth MCP joints in 26 patients with SLE - 9 classified as having rhupus syndrome, 10 as having Jaccoud’s arthropathy (JA) and 7 as having non-deforming non-erosive (NDNE) arthritis - were subdivided into areas and bilaterally evaluated for the presence of bone erosion by CT and US. On CT, erosion volume was scored according to the outcome measures in rheumatology-rheumatoid arthritis magnetic resonance imaging (OMERACT-RAMRIS) score. On US, erosions were semi-quantitatively scored 0–3 according to scoring by ultrasound structural erosion (ScUSSe) systems.

**Results:**

Erosions were detected by CT in 92/728 areas (12.6 %) and by US in 43/728 areas (5.9 %). Sensitivity, specificity and accuracy of US overall was 36 %, 98 % and 90 % compared with 57 %, 98 % and 93 % in the dorsal and lateral aspects of the second and fifth MCP, which were identified as areas with the best US reliability. Adding wrist joints would capture a larger number of erosions without affecting the accuracy. US detected 90.0 % of CT erosions with bone volume loss >20 % and 51.2 % of erosions with bone volume loss >10 %. Patients with rhupus had a greater number of larger erosions than those with JA or NDNE arthritis, with prevalent involvement of the MCP joints. Overall reliability of US in detecting bone erosions was moderate for rhupus syndrome (0.55) and JA (0.58), but poor for NDNE arthritis (0.10).

**Conclusion:**

US had moderate sensitivity and excellent specificity for detection and semi-quantitative assessment of bone erosions in SLE.

## Background

Arthritis in systemic lupus erythematosus (SLE) is usually non-erosive on x-ray, even in the 5–15 % of patients with long-standing disease who develop hand and foot deformities as hallmarks of Jaccoud’s arthropathy (JA) [1, 2]. An exception is represented by rhupus syndrome, which affects fewer than 5 % of patients and is characterized by erosions on radiographs of the hands and feet, persistent synovitis and a high titer of anti-cyclic citrullinated peptide (aCCP) antibodies, and may be considered an overlap between rheumatoid arthritis (RA) and SLE [3, 4]. The increased number of reports on rhupus syndrome may have also influenced the 2012 Systemic Lupus International Collaboration Clinics (SLICC) classification criteria statement that some SLE arthritis may, in fact, be erosive [5]. Recent ultrasonography (US) studies show an unexpected burden of erosive damage, challenging the classification of SLE arthritis as non-erosive, and also demonstrate that US is more sensitive than conventional radiography in detecting bone erosions in SLE [6–11]. However, great variability in erosion rates (from 2 % to 41 %) has been reported, probably because different joint sites were included in the US protocols, and most studies did not clearly separate patients with rhupus syndrome from other patients with SLE [12]. US-detected bone erosions are associated with future appearance of erosions on radiography and worse prognosis in patients with RA [13]. In contrast to RA, the nature and progression of US-detected erosions in SLE is poorly understood and whether erosions detected by US or radiography imply a different course is yet unknown and deserves further prospective studies [14].

A semi-quantitative scoring system has been developed to assess the size of US bone erosions [15] and has been applied in RA [16, 17] but not in SLE. Furthermore, the hand and wrist joints in which US performs better in detecting bone erosions have never been investigated in patients with SLE. US is increasingly being used in daily practice and increasingly features in scientific reports for the assessment of patients with SLE arthritis, therefore, it is of great interest to evaluate the reliability and performance of US for erosion detection in this disease. Computed tomography (CT) has been shown to be the gold standard for detecting erosions and therefore could be considered the best reference method [18, 19].

The aims of the present study were, first, to evaluate the sensitivity, specificity and accuracy of high-resolution US in different areas of the wrist and hand joints in patients with SLE using multislice CT as the gold standard reference method and, second, to evaluate the accuracy of US and compare erosive features in the different subtypes of SLE arthritis.

## Methods

### Study cohort

Imaging studies from 26 Caucasian patients with SLE participating in a larger prospective study (the Systemic Lupus Erythematosus Musculoskeletal Manifestation Study (SLEMMS)) were used to compare the detection of erosions in the wrist and metacarpophalangeal (MCP) joints using CT and US. All patients were diagnosed as suffering with SLE according to the 1997 American College of Rheumatology (ACR) classification criteria and were further classified as suffering with rhupus syndrome, JA or non-deforming and non-erosive (NDNE) arthritis.

After classification according to the subtypes of SLE arthritis, patients were consecutively enrolled into three different subgroups: (1) rhupus syndrome, which was defined by the presence of bone erosions on x-ray and by fulfillment of classification criteria for both RA and SLE; (2) JA, which was defined as the presence of one or more of the following reducible or fixed hand deformities: MCP subluxation, ulnar drift, swan neck or boutonniere fingers and Z thumbs in the absence of x-ray-detected erosions and (3) the remaining patients, who were classified as having NDNE arthritis.

Study inclusion criteria were: (a) ≥18 years old, (b) past or present musculoskeletal inflammatory involvement and (c) capable of giving consent. Exclusion criteria were: (a) women who were pregnant or breastfeeding and (b) women of child-bearing potential, or men whose partners were women of childbearing potential and who were unwilling to use effective contraception within ± 1 month of undergoing CT.

### US and CT acquisition and assessment

The ulnar head, radiocarpal and second to fifth metacarpal heads were bilaterally examined by CT and US in 26 patients, and were evaluated for the presence of bone erosion in 14 areas on each side: (a) a dorsal and lateral scan of the ulnar head and radiocarpal joint (including the distal radial epiphysis, scaphoid and lunate bones); (b) the dorsal, lateral and palmar aspect of the second and fifth metacarpal head and (c) the dorsal and palmar aspect of the third and fourth metacarpal head. US and CT images were independently assessed by a rheumatologist (AG) and a radiologist (LS), respectively, blinded to clinical and other imaging data.

CT was performed on a 16-multi-detector-row CT scanner (Philips Brilliance, Philips, Eindhoven Netherland). Parameters used were voxel size 0.5 mm × 0.5 mm × 0.5 mm (isotropic voxel), pitch 0.5, slice spacing 0.5 mm, overlap 50 %, 120 kV and 280 mAs. Images were reconstructed in the coronal and axial planes with a slice thickness of 0.5 mm. Erosion was defined as a sharply juxta-articular marginated bone lesion, visible in two planes, and with a cortical break seen in at least one plane. Moreover, CT images were scored for erosions according to the outcome measures in rheumatology (OMERACT)/rheumatoid arthritis magnetic resonance imaging score (RAMRIS) method [20, 21]. According to RAMRIS, bone erosion was graded by percentage volume (0–10, by 10 % volume increments) of the assessed bone. Each bone (in the wrists, the carpal bones, distal radius, distal ulna and metacarpal bases; in the MCP joints, the metacarpal heads and phalangeal bases) is scored separately. The scale is 0–10, based on the proportion of eroded bone compared to the “assessed bone volume” judged on all available images (0 = no erosion, 1 = 1–10 % of bone eroded, 2 = 11–20 % eroded ; 3 = 21–30 % eroded, and so on). For metacarpal and phalangeal bone the “assessed bone volume” is from the articular surface to a depth of 1 cm, and in carpal bones it is the whole bone. In total 23 bone sites are evaluated, leading to a total RAMRIS erosion score ranging between 0 and 230 for each hand (80 for the MCP and 150 for the wrist joints).

US examinations were performed using a Logiq9 (General Electric Medical Systems, WI, USA) equipped with an 8 − 15 MHz (4D16L) volumetric probe (lateral resolution = 0.3 mm, axial resolution = 0.1 mm). Patients were seated with their hands positioned on an examining table in supination for the volar scans and pronation for the dorsal scans, with the wrist in a neutral position. Joints were bilaterally evaluated using a multi-planar scanning technique. According to OMERACT, US bone erosion was defined as an intra-articular discontinuity of the bone surface that is visible in two perpendicular planes [22]. In addition to this categorical assessment (present/absent), erosions were scored on a 0–3 scale in each joint area according to the maximal length (0 = no erosion, 1 = <2 mm, 2 = 2–3 mm, 3 = >3 mm) as suggested by the scoring ultrasound structural erosion (ScUSSe) system [15].

### Statistical analysis

Relevant features were reported as mean ± standard deviation (SD) or median (25th to 75th percentiles) for normal or non-normal distributed variables, according to the Kolmogorov-Smirnov test, and number with the corresponding percentage. Student’s *t* test, or the Mann-Whitney test if necessary, were used to compare the distribution of quantitative variables in different subtypes of SLE arthritis. Two-tailed *p* values <0.05 were considered significant.

The sensitivity, specificity and accuracy (or exact agreement) of US for detecting erosion in the hand and wrist joints in patients with SLE were calculated using CT as the reference method. This was repeated for each joint and for each subtype of SLE arthritis. The agreement between CT and US was additionally evaluated by weighted k values and by the proportion of US erosions confirmed by CT. Reliability analysis of CT and US assessment was evaluated by inter-observer agreement rates (k values) with a second radiologist (AB) and another rheumatologist (MP), respectively, both blinded to the other imaging data. The radiologist made an assessment on stored CT images, whereas the rheumatologist performed an independent UD examination on the same day. A weighted k value of 0–0.20 was considered poor, 0.21–0.40 fair, 0.41–0.60 moderate, 0.61–0.80 good and 0.81–1.00 excellent; the 95 % confidence interval (CI) was also calculated. Statistical analyses were performed using the software package MedCalc 15.0 (MedCalc Software, Mariakerke, Belgium).

## Results

The mean age of the enrolled patients was 49.4 ± 15.5 years with mean disease duration of 16.0 ± 8.2 years. Among the enrolled patients, 9 out of 26 (34.6 %) were classified as having rhupus syndrome, 10/26 (38.5) as having JA and 7/26 (26.9 %) as having NDNE arthritis. The patients’ demographic data are presented in Table 1.

**Table 1:** Baseline demographic data and cumulative clinical and serological data of the study cohort

	SLE (n = 26)	Rhupus syndrome (n = 9)	Jaccoud’s arthropathy (n = 10)	NDNE (n = 7)
Age (mean ± SD)	49.4 ± 16.0	41.1 ± 5.7	61.3 ± 14.3	43.1 ± 16.5
Disease duration (mean ± SD)	15.6 ± 8.2	13.7 ± 6.7	21.7 ± 8.5	10.8 ± 4.5
Gender, female, *n*	26	9	10	7
Cumulative 1997 ACR criteria				
Malar rash	12 (46.2)	5 (55.5)	4 (40.0)	3 (42.8)
Discoid rash	2 (7.7)	0	1 (10.0)	1 (14.3)
Photosensitivity	4 (15.4)	1 (11.1)	2 (20.0)	1 (14.3)
Oral/nasopharyngeal ulcers	2 (7.7)	0	1 (10.0)	1 (14.3)
Serositis	15 (57.7)	4 (44.4)	8 (80.0)	3 (42.8)
Renal disorders	5 (19.2)	0	2 (20.0)	3 (42.8)
Neurologic disorders	2 (7.7)	0	2 (20.0)	0
Hematological disorders	14 (53.8)	5 (55.5)	7 (70.0)	2 (28.6)
ANA	26 (100)	9 (100)	10 (100)	7 (100)
Anti-dsDNA	18 (69.2)	6 (66.7)	8 (80.0)	4 (57.1)
Antiphospholipid antibodies	7 (26.9)	3 (33.3)	3 (30.0)	1 (14.3)
Other laboratory findings				
Anti-Ro/SSA	14 (53.8)	4 (44.4)	5 (50.0)	5 (71.4)
Anti-La/SSB	2 (7.7)	0	1 (10.0)	1 (14.3)
Anti-RNP	2 (7.7)	0	0	2 (28.6)
Anti-Sm	2 (7.7)	0	0	2 (28.6)
Rheumatoid factor	11 (37.9)	7 (77.8)	1 (10.0)	3 (42.8)
Anti-CCP	7 (26.9)	6 (66.7)	0	1 (14.3)
SLICC/damage index (mean ± SD)	1.4 ± 1.3	1.4 ± 0.5	2.6 ± 1.3	0.6 ± 0.5
Medication received^a^				
Total corticosteroid dose >50 g	15 (57.7)	3 (33.3)	10 (100)	2 (28.6)
Antimalarial agents	25 (96.1)	8 (88.9)	10 (100)	7 (100)
Methotrexate	20 (76.9)	9 (100)	6 (60.0)	5 (71.4)
Azathioptine	7 (26.9)	1 (11.1)	4 (40.0)	2 (28.6)
Micophenolate	4 (15.4)	1 (11.1)	1 (10.0)	2 (28.6)
Rituximab	8 (30.7)	4 (44.4)	2 (20.0)	2 (28.6)
Belimumab	1 (3.8)	0	0	1 (14.3)
Anti TNF-alpha	3 (11.5)	3(33.3)	0	0
Anti IL-6	2 (7.7)	1 (11.1)	1 (10.0)	0

Unless otherwise specified, values are the number of patients (values in brackets are percentage). ^a^Includes medication ongoing at enrollment and past treatment. Antiphospholipid antibodies included lupus anticoagulant, anticardiolipin antibodies IgG and IgM, beta2-glicoproteinI IgG and IgM. Anti-extractable nuclear antigens (SSA, SSB, Sm, RNP, Rheumatoid factor and anti-cyclic citrullinated peptide antibodies (antiCCP) were tested by ELISA. Antinuclear antibodies (ANA) were tested by IIF, using Hep2 cell substrate; positivity was defined as a titer ≥1:320. Anti-double-stranded DNA (Anti-dsDNA) antibodies were tested by Farr assay. *SLE* systemic lupus erythematosus, *NDNE* non-deforming non-erosive, *ACR* American College of Rheumatology, *SLICC* Systemic Lupus International Collaboration Clinics

Inter-observer agreement rates between the two sonographers were excellent for erosion detection (k value = 0.86, 95 % CI 0.65, 0.98) and that between the two radiologists was similar (k value = 0.84, 95 % CI 0.64, 0.97).

### Detection of erosions by CT and US

Erosions were detected by CT in 92 out of 728 areas (12.6 %) and by US in 43/728 areas (5.9 %) (Table 2). With CT as the reference method, US had overall sensitivity of 35.9 % and specificity of 98.7 % for the detection of bone erosions in the hands in patients with SLE. The exact agreement was 90.8 % with a moderate k value of 0.50 (95 % CI 0.36, 0.63). Values differed according to the areas assessed, with the US having the highest sensitivity in the dorsal and lateral aspect of the second and fifth metacarpal heads (Table 2). When only the second and fifth MCP areas were included the overall sensitivity of US increased to 57.1 %, the specificity was 97.8 % and accuracy 93.3 % (k value 0.66, 95 % CI 0.50, 0.83). If the ulnar and radiocarpal areas were added to the second and fifth MCP areas the sensitivity remained moderate (45.7 %) with excellent specificity (98.4 %), excellent accuracy (91.3 %) and good reliability (k value 0.63, 95 % CI 0.50, 0.75). Investigating the volar aspect of MCP heads did not add value to the US examination, as erosions in these quadrants were rare (0.9 %).

**Table 2:** Distribution of erosions in different region of interest according to CT and US, and sensitivity, specificity, accuracy and k values for high-resolution US in detecting erosion (grading from 0 to 3) at different joint sites using CT as the reference method

	Quadrants	CT-detected erosions	US-detected erosions	Sensitivity (%)	Specificity (%)	Exact agreement (%)	k values (95 % CI)
Second metacarpal head	156	27 (17.3)	21 (13.5)	51.9	95.3	87.8	0.63 (0.43 to 0.83)
Dorsal	52	16 (30.8)	13 (25.0)	56.3	88.9	78.8	0.51 (0.26 to 0.75)
Lateral	52	11 (21.2)	8 (15.4)	54.5	95.1	86.5	0.77 (0.54 to 1.00)
Volar	52	0	0	NC	NC	NC	NC
Third metacarpal head	104	15 (14.4)	4 (3.8)	20.0	98.9	87.5	0.26 (0.03 to 0.48)
Dorsal	52	14 (21.2)	4 (15.4)	21.4	97.4	76.9	0.24 (0.02 to 0.46)
Volar	52	1 (1.9)	0	0	100	98.1	0 (0.00 to 0.00)
Fourth metacarpal head	104	7 (6.7)	1 (0.9)	14.3	100	94.2	0.19 (-0.12 to 0.51)
Dorsal	52	6 (11.5)	1 (1.9)	17.0	100	90.4	0.08 (-0.09 to 0.26)
Volar	52	1 (1.9)	0	0	100	98.1	0 (0.00 to 0.00)
Fifth metacarpal head	156	8 (5.1)	6 (3.8)	50.0	100	96.3	0.77 (0.66 to 0.89)
Dorsal	52	6 (11.5)	4 (7.7)	66.7	100	96.2	0.73 (0.56 to 0.90)
Lateral	52	2 (3.8)	2 (3.8)	100	100	100	0.85 (0.74 to 0.97)
Volar	52	0	0	NC	NC	NC	NC
Radiocarpal	104	18 (17.3)	6 (5.8)	33.0	100	88.5	0.57 (0.30 to 0.83)
Dorsal	52	15 (28.8)	4 (7.7)	26.7	100	78.8	0.48 (0.16 to 0.80)
Lateral	52	3 (5.7)	2 (3.8)	66.7	100	98.1	0.79 (0.39 to 1.0)
Ulnar head	104	17 (16.3)	5 (4.8)	23.5	98.9	86.5	0.29 (0.05 to 0.54)
Dorsal	52	13 (25.0)	4 (7.7)	23.1	97.4	78.8	0.23 (-0.03 to 0.50)
Lateral	52	4 (7.7)	1 (1.9)	25.0	100	94.2	0.38 (-0.15 to 0.91)
Total	728	92 (12.6)	43 (5.9)	35.9	98.7	90.8	0.50 (0.36 to 0.63)

Unless otherwise specified, values are the number of patients (values in brackets are percentage). *CT* computed tomography, *US* ultrasound, *NC* not computable

There were no major differences in the results for the dominant and non-dominant hand. CT detected erosions in 46 areas in both hands. The exact agreement between CT and US was 91.5 % for the dominant hand (k value = 0.50, 95 % CI 0.32, 0.69) and 90.1 % for the non-dominant hand (k value = 0.49, 95 % CI 0.30, 0.68).

### Bone volume loss on CT and US

In 8 out of the 43 areas (18.6 %) with US-detected erosions, the US erosions were not confirmed by CT. These were located at the second MCP (n = 6), third MCP (n = 1) and the ulnar head (n = 1) and all of them were scored 1 using the ScUSSe. On reassessment of these areas the major confounding reasons were cortical irregularities, osteophytes and bone surface indentation at the metacarpal neck. In one patient an erosion was detected on reassessment by CT.

US detected 51.2 % of areas with erosions where the bone volume loss detected by CT was >10 %, irrespective of the localization. When only the areas with the best US reliability were included, that proportion increased to 70.0 % (Table 3). There was only one large erosion at the third MCP (grade 3) that was detected on CT but not identified by US.

**Table 3:** Erosions detected by CT in joint quadrants and semi-quantitatively assessed according to the RAMRIS erosion score, and proportion of erosions detected by US

RAMRIS erosion score	Quadrants with CT erosion (*n*)	US-detected erosions (%)
All areas		
Grade 1 (1–10 %)	51	27.5
Grade 2 (11 − 20 %)	31	41.9
Grade 3 − 10 (21–100 %)	10	90.0^a^
Areas with best US reliability^b^		
Grade 1 (1 − 10 %)	15	53.3
Grade 2 (11 − 20 %)	15	60.0
Grade 3 − 10 (21 − 100 %)	5	100.0

^a^Only one large computed tomography (CT)-detected erosion (grade 3) at the third metacarpophalangeal join (MCP) was not identified by ultrasound (US) because the cortical break was not accessible to US. ^b^Second MCP and fifth MCP quadrants. *RAMRIS* rheumatoid arthritis magnetic resonance imaging score

### Subtypes of SLE arthritis

**Fig. 1:**
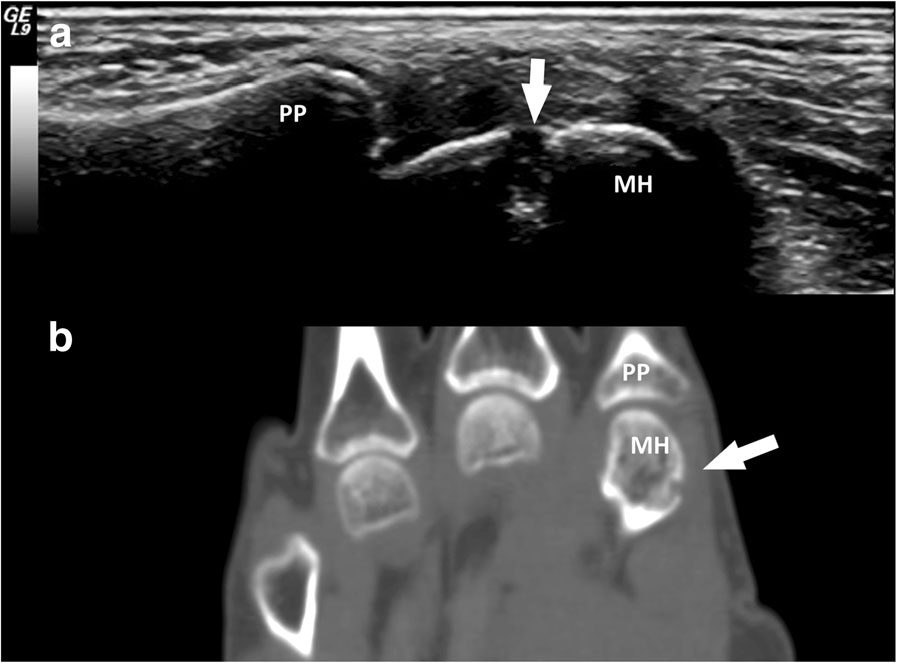
Bone erosions in a patient with rhupus syndrome. Longitudinal ultrasound image of the second metacarpophalangeal joint lateral area showing a grade-2 scoring ultrasound structural erosion (*arrow*) with sharp margins (**a**) corresponding to a grade-2 rheumatoid arthritis magnetic resonance imaging score erosion (*arrow*) visible in the computed tomography coronal scan of the hand (**b**). *MH* second metacarpal head, *PP* second proximal phalanx

Erosions were detected by US in 9/9 patients with rhupus (100 %), 5/10 patients with JA (50.0 %) and 3/7 patients with NDNE (42.8 %). Patients with rhupus syndrome had larger numbers of erosions and higher ScUSSe grades of erosion than patients with JA and NDNE (Table 4). The most frequent localization of US-detected erosion in rhupus syndrome was at the second MCP (90.0 %) (Fig. 1) and the fifth MCP joints (44.4 %) and the wrist joint (44.4 %). The second MCP and wrist joints were equally affected by erosion in JA (30.0 %) and NDNE arthritis (28.6 %).

**Table 4:** Main characteristics and distribution of US and CT abnormalities in patients with different subtypes of SLE arthropathy

	Rhupus (n = 9)	JA (n = 10)	NDNE (n = 7)	Rhupus vs JA (*p* value)	Rhupus vs NDNE (*p* value)	JA vs NDNE (*p* value)
Age, years (mean ± SD)	41.0 ± 5.7	61.3 ± 14.3	43.1 ± 16.4	**0.001**	0.765	**0.035**
Disease duration, years (mean ± SD)	13.7 ± 6.6	21.7 ± 10.7	10.8 ± 4.1	**0.035**	0.298	**0.003**
Rheumatoid factor	7 (77.8 %)	2 (20.0 %)	2 (28.5 %)	**-**	**-**	-
Anti-CCP	6 (66.7 %)	0 (0)	1 (14.2 %)	**-**	**-**	-
US erosion number (median (25th to 75^th^ percentile))	3.0 (1.7–4.2)	0.5 (0–2.0)	0 (0–1.0)	**0.019**	**0.004**	0.569
US quadrants with erosion	27 (10.7 %)	12 (4.3 %)	4 (2.0 %)	**-**	**-**	-
ScUSSe total (median (25th to 75^th^ percentile))	4.0 (2.5–6.0)	1.0 (0–3.0)	0 (0–1.0)	**0.021**	**0.003**	0.535
Grade 1 (<2 mm)	16^a^	10^a^	4^a^	**-**	**-**	**-**
Grade 2 (2–3 mm)	9^a^	1^a^	0^a^	**-**	**-**	**-**
Grade 3 (>3 mm)	2^a^	1^a^	0^a^	**-**	**-**	**-**
CT erosion number (mean ± SD)	14.0 ± 9.7	5.7 ± 5.1	4.7 ± 4.5	**0.050**	**0.030**	0.543
RAMRIS (mean ± SD)	21.6 ± 18.2	7.4 ± 7.0	5.1 ± 5.0	0.053	**0.029**	0.448
RAMRIS MCP (median (25th to 75^th^ percentile))	6.0 (4.5–10.2)	1.0 (0–2.0)	0 (0–1.5)	**0.013**	**0.009**	0.353
RAMRIS wrist (median (25th to 75^th^ percentile))	8.0 (10.2–16.2)	3.5 (0–11.0)	3.0 (0.5–6.7)	0.111	0.057	0.694
Grade 1 (1–10 %)	24^a^	21^a^	6^a^	**-**	**-**	**-**
Grade 2 (11–20 %)	19^a^	6^a^	6^a^	**-**	**-**	**-**
Grade 3 − 10 (21–100 %)	7^a^	1^a^	2^a^	**-**	**-**	**-**

Numbers in bold indicate statistical significance for p <0.05. Boxes are empty where statistics was not applied

^a^Absolute numbers referring to joint quadrants. *JA* Jaccoud’s arthropathy, *NDNE* non-deforming non-erosive, *Anti-CCP* anti-cyclic citrullinated peptide, *US* ultrasound, *ScUSSe* scoring ultrasound structural erosion, *CT* computed tomography, *RAMRIS* rheumatoid arthritis magnetic resonance imaging score, *MCP* metacarpophalangeal joint

Using CT as the reference method, the overall reliability of US in detecting bone erosions was moderate for rhupus syndrome (k value = 0.55, 95 % CI 0.38, 0.71) and JA (k value = 0.58, 95 % CI 0.32, 0.83), whereas it was poor for NDNE arthritis (k value = 0.10, 95 % CI 0.05, 0.26).

Erosions were detected by CT in 9/9 patients (100 %) with rhupus syndrome, 8/10 patients (80.0 %) with JA and 5/7 patients (71.4 %) with NDNE arthritis. The median RAMRIS score for erosions was 10.0 (1.0–15.0). CT-detected erosions were more frequently identified in the wrist (in rhupus syndrome 9/9 (100 %), in JA 7/10 (70.0 %) and in NDNE arthritis 5/7 (71.4 %) than in the MCP joints (in rhupus syndrome 8/9 (88.8 %), in JA 6/10 (60.0 %) and in NDNE arthritis 2/7 (28.6 %). The burden of damage calculated by RAMRIS erosion score was differently distributed, being significantly higher at the MCP joints in patients with rhupus syndrome than in patients with JA and NDNE arthritis (Table 4). No major differences were found between JA and NDNE arthritis.

## Discussion

This is the first study to date assessing the bone erosive burden in patients with SLE by using semi-quantitative US and CT scoring systems and defining the accuracy of US in detecting bone erosions in the hands and wrists of patients with SLE, using CT as the reference method. In the present study US had very high specificity (98.7 %) and fair sensitivity (35.9 %) for detecting erosions when compared to CT. US had fair sensitivity of 23.5 % in the wrist joint but when only the MCP joints were included in the analysis, the sensitivity increased to 40.4 %, and by further restricting analysis to the second and fifth MCP joints, which are considered the areas with the best US reliability, sensitivity increased to 57.1 %.

These findings are in agreement with the results from CT studies performed on MCP joints in patients with RA, showing overall moderate sensitivity of 42–44 % [16, 18]. However, Dohn et al. reported sensitivity of 71 % when investigating areas with the best US accessibility [16]. The lower US sensitivity observed in the present study could be due to poor ability to detect small erosions in patients with non-rhupus SLE, compared to that observed in RA. In magnetic resonance imaging (MRI) studies in SLE, MCP joint erosions have been detected in 47–61 % of patients and wrist joint erosions have been detected in 82 − 99 % of patients [9, 23–25], which roughly corresponds to our results, with 61.5 % and 80.7 % of patients with CT-detected erosions detected in the MCP and wrist joints, respectively. Compared with CT and MRI, the performance of US was confirmed to be worse for detection of erosions, especially when assessing the wrist. In fact, in this and previous US studies [6–9] the incidence of wrist erosions was lower (4 − 20 %) than that of MCP erosions (20 − 41 %), in contrast to what has been shown by CT and MRI.

The distribution of erosive damage could be of particular interest in differentiating between subtypes of SLE arthritis. Tani and colleagues [26] showed that the cumulative burden and distribution of MRI-detected erosions in rhupus syndrome were similar to those in RA and significantly higher than in non-rhupus SLE. We further characterized these features proving that both US and CT detected a higher proportion of grade-2 and grade-3 erosions in rhupus syndrome than in patients with JA and NDNE arthritis. Noteworthy, patients with rhupus syndrome had a significantly higher burden of erosive damage in the MCP joints than those with JA and NDNE arthritis detected by both CT and US, but no significant differences were found in the wrist joints.

Along with these novel observations, we provide data on the largest series published so far of US assessment of the hands in JA. By contrast to our previous experience, reporting US erosions in 16.7 % (1 out of 6) of patients with JA [6], in the present study the prevalence of erosions detected by US and CT was 50.0 % and 80.0 %, respectively. This is similar to that reported by Sa Ribeiro and colleagues [27], showing MRI-detected MCP erosions in 50.0 % of patients with JA. These contrasting results could be due to the different joint sites investigated in our previous study [6], which included areas with low US sensitivity such as the third MCP joint and the wrist, and, by contrast, did not assess the fifth MCP joint.

**Fig. 2:**
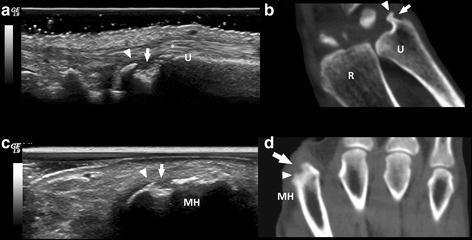
Bone erosions in patients with Jaccoud’s arthropathy. Longitudinal ultrasound (US) image of the lateral ulnar head area (*U*) showing a grade-2 scoring ultrasound structural erosion (ScUSSe) (*arrow*) with a hook-shaped deformity (*arrow head*) (**a**). *U* ulnae, *R* radial, *extensor carpi ulnaris tendon. Coronal computed tomography (CT) scan of the wrist showing a grade-4 rheumatoid arthritis magnetic resonance imaging score (RAMRIS) erosion (*arrow*) confirming the hook-shaped deformity (*arrow head*) with a bony sclerotic margin (**b**). Transversal US view of the second metacarpophalangeal (MCP) dorsal area showing a grade-3 ScUSSe hook-shaped (*arrow head*) erosion (*arrow*) (**c**) corresponding to a grade 3 RAMRIS score erosion (*arrow*) with hook-shaped deformity (*arrow head*) visible in the CT coronal scan of the hand (**d**). *MH* second metacarpal head

Moreover, the erosive damage in both JA and NDNE arthritis did not resemble that seen in rhupus syndrome and RA. Previously published studies do not report “hook” erosions in RA [15–21, 23], which are instead considered a hallmark of JA [2, 24, 28]. Although the analysis of hook erosions in the different subtypes of SLE arthritis was not an objective of this study, we found that only the large erosions detected by US and CT in patients with JA had a well-defined hook-shaped deformity with a sclerotic margin (Fig. 2) replicating, in fact, the hook erosions rarely seen on radiographs [28]. Erosions observed in patients with NDNE arthritis were actually fewer and smaller than in rhupus syndrome and JA, which could explain why the sensitivity and reliability of US in this group of patients was poor using CT as the gold standard reference method. Further studies, aimed at comparing erosions in the different subtypes of SLE arthritis, are needed, as our findings might be related to the small sample size, representing a major limitation; however, this was a secondary objective of the study.

The present study has some other limitations. First, ScUSSe and RAMRIS were not specifically developed for use in patients with SLE and are based on different definitions and erosion scoring, which may have influenced the agreement between the two methods. Nevertheless, using existing scoring systems makes the results comparable to other RA and SLE studies. Second, the rate of erosion reported in present study may have been affected by differences in and time to initiation of concomitant and prior treatment. It is worth noting that the only significant treatment difference between the subgroups of patients was a higher cumulative dose of steroid registered in patients with JA, which has been recently envisaged as a major cause of joint laxity and development of deformities in patients with SLE [14]. Finally, a comparison of US, CT, and conventional radiography was not performed. This could be considered a limitation; however, radiography has low sensitivity for the detection of bony erosive damage in SLE [1, 6, 14, 29] and for classification of patients [1, 25, 26], and therefore, it should be limited to first-line assessment and for differential diagnosis.

## Conclusions

Overall, the most important message to emerge from the present study is that US detection of bone erosions in patients with SLE has very high specificity and also moderate sensitivity when restricted to the second and fifth MCP joints, which are the joints that can be assessed with high reliability. Adding the wrist joint will enhance the ability to identify a greater number of erosions, preserving good reliability and only weakly affecting the accuracy. Even though it is still not possible to make an early differentiation between SLE arthritis subtypes using US exclusively, the high specificity of US for detecting bone erosions suggests that it may play a role in the future in early classification of different SLE arthritis subtypes. A classification system could be particularly helpful in order to provide targeted therapies to patients at risk of developing erosive or deforming arthritis as a major cause of disability. An increasing number of scientific reports suggest, in fact, that patients with rhupus syndrome may take advantage of anti-CD20 [30] or anti-CTL4 [31] biologic drugs, whereas TNF-alpha blockers are discouraged [32]. On the other hand, JA has been suggested to be a late complication of NDNE arthritis, characterized by high IL-6 and C-reactive protein and sub-clinical inflammation [11, 14, 33], and therefore, may benefit from early anti-BLyS [34] or anti-IL-6 treatment. In conclusion, further studies aimed at outlining a classification system and shedding some light on the mechanisms underlying the development and progression of US-detected erosions in SLE must assess the erosive changes using a semi-quantitative scoring system, and must focus on the second and fifth MCP and wrist joints in order to ensure the highest sensitivity and specificity and capture the higher burden of erosive damage.

## Abbreviations

aCCP: anti-cyclic citrullinated peptide
ACR: American College of Rheumatology
BLyS: B-lymphocyte stimulator
CD20: cluster differentiation 20
CI: confidence interval
CT: computed tomography
CTL4: cytotoxic T-lymphocyte antigen 4
ELISA: enzyme-linked immunosorbent assay
IL-6: interleukin 6
JA: Jaccoud’s arthropathy
kV: kilovolt
mAs: milliamperes
MCP: metacarpophalangeal
mm: millimeter
MRI: magnetic resonance imaging
NDNE: non-deforming non-erosive
OMERACT: Outcome Measures in Rheumatology
RA: rheumatoid arthritis
RAMRIS: rheumatoid arthritis magnetic resonance imaging score
ScUSSe: scoring ultrasound structural erosion
SD: standard deviation
SLE: systemic lupus erythematosus
SLEMMS: Systemic Lupus Erythematosus Musculoskeletal Manifestation Study
SLICC: Systemic Lupus International Collaboration Clinics
TNF: tumor necrosis factor
US: ultrasonography
